# Treatment of Scarlet Fever

**Published:** 1854-07

**Authors:** Geo. H. Hubbard


### ECLECTIC DEPARTMENT.

---

### *Treatment of Scarlet Fever.*

BY GEO. H. HUBBARD, M. D.

The vexed question of contagion we leave to be discussed by those who like, and will confine ourself to a short exposition of the principles on which we found a plan of treatment which has in our hands been eminently successful.

We consider scarlatina a contagious fever, to be classed with the other contagious exanthemata, less contagious than small pox, more so than typhoid (continued) fever, or erysipelas. Like all other eruptive fevers, it sometimes springs up where we have no reason to attribute it to contagion or infection, and appears, so far as our knowledge extends, to be self-developed. This may terminate as a sporadic case, or may be the starting point of a wide-spread epidemic; depending upon atmospheric vicissitudes and individual predispositions very imperfectly understood.

*Its period of incubation* may be considered from four to eight days. Dating from the first feeling of illness, the rash should begin to appear in twenty-four hours, and should begin to fade in four or five days from its first appearance. This may be considered the regular course of the disease, when it does its perfect work unembarrassed by accidents or injurious medication. But the number of cases in which the true order of nature is followed is small, compared to those which are thrown more or less from their natural course by accidental vicissitudes.

On an early and perfect eruption depends the perfection of the disease and the safety of the patient. Not that there is no danger from the accidental local lesions so frequent during its progress, but that there is no safety when the eruption is imperfectly developed, or retroceded, or prematurely faded, To allow nature to produce this eruption at the proper time, rendering aid when she is incapable, and withholding officious treatment, is the duty of the true physician.

No physician who has witnessed the graver forms of this disease can have overlooked the fearfully severe struggle which is going on from the first invasion to the perfection of the eruption. In most cases this struggle terminates in the triumph of nature and an amelioration of the sufferings of the patient upon the appearance of the rash; but the powers of the patient may not be sufficient, and the rash may fail to appear from three causes,—a hyperæmic condition requiring antiphlogistic remedies; an anæmic, demanding stimulants, and the irritated, which threatens the life of the child, from the destructive impression produced on the nervous centres through sympathy with the cutaneous disease.

Of the first of these conditions we propose to say but little.—Phlogosis has been the bane and depletion the remedy of medical writers for the last half century, and the disease under consideration has received its full share of antiphlogistic attention. We have only to say we rarely find it necessary to resort to antiphlogistic treatment in scarlet fever. An emetic or laxative at the onset, which are very likely to be abused, and which will often be anticipated by the spontaneous effort of nature, are the principal remedies of this class found necessary in several epidemics of this disease which we have treated.

The anæmic condition, or that in which the vital powers were inadequate to perfect the eruption, has been that which has oftenest required our interference. In cases of this character we find a rapid, weak and small pulse, skin dry, not very hot, with little or no appearance of the rash after the expiration of twenty-four hours from the invasion of the disease. The want of power seems most apparent in the vascular system, more especially the vessels of the cutaneous surface; and the great indication is to aid nature in her effort to throw out the rash by those remedies which determine to increased action of the cutaneous vessels. In a word, that class of remedies which we call diaphoretics.

But in choosing from this class we are to keep in view the condition of our patient, and choose that particular article which will act in harmony with the debilitated condition of the system; thus the Pot. Tart. of Antimony would be manifestly injurious; for, although a powerful diaphoretic, it is a decided anti-phlogistic, and acts powerfully to depress the vital powers. Ipecac is less objectionable, and in many cases and in some epidemics is the proper remedy; so often so, that nearly all our systematic writers recommend "a gentle emetic at the outset," as ordinarily safe and proper. This we consider as altogether too sweeping a suggestion; and that the administration of an emetic should be governed by the result of a careful inquiry into the condi tion of our patient; and only after a careful consideration of the question, Will the action of an emetic do most good by the tendency to promote cutaneous action, or harm by its revulsive effect, in determining from the skin to the gastro enteric mucous surface ?

The diaphoretic which has proved in our hands the most safe and reliable in treating this class of cases, is the liquid acetate of ammonia. This we prepare extemporaneously, by neutralizing good cider vinegar with pure carbonate of ammonia; and as the carbonate is of itself little if any inferior as a diaphoretic to the acetate, we prefer a slight excess of this in the solution, and where the debility is very marked we allow this excess to be considerable.

This liquid acetate of ammonia is a diaphoretic, which produces its effect by a stimulant rather than an antiphlogistic action; and although an observer unfamiliar with this disease would perhaps hesitate in prescribing stimulants when the generel symptoms are so decidedly febrile, yet an enlightened experience will show him that on them often depends the safety of his patient.

In many cases of this class the Dover powder is an invaluable remedy; and is a favorite prescription in ordinary cases is this alternating with the acetate of ammonia, which in cases of ordinary severity will yield all the stimulant, diaphoretic and anodyne influence demanded; but we frequently meet with cases where as free an exhibition of the ammonia as a sound common sense will allow falls short of proving adequate to the wants of our case, and the use of the warm bath, and direct stimulation with pure spirit, is necessary to the safety of our patient.

Young children are often in imminent danger, both before and after the full appearance of the eruption, from the severity of the constitutional irritation which their immature nervous organization is unable to withstand. Frequently on the second day of the disease we may find our little patient showing little or no rash, but incessantly changing its position; tossing about the bed to such an extent as to render it impossible to keep it covered. The same condition may arise from a retrocession of the rash after its full appearance. Here a mild or expectant course would be sure to end in the death of the little sufferer; but a cool and judicious application of remedial measures will rarely fail. The warm bath and opium are our main hopes in these cases. The first tends powerfully to aid nature in perfecting the eruption; the second is indispensably necessary to keep the patient alive till this is accomplished, and bad luck to the timid practitioner who stops before quieting his patient, because he has given large and repeated doses of some anodyne without apparent effect.

In some of these cases a child of a few years will require as free an exhibition of opium as we would be willing in ordinary cases to direct for a much older patient; but the child must rest or its life will soon pass away; and the judicious physician who finds his patient in this state will not leave it till he has by frequent repetitions of a moderate dose brought it decidedly under its influence, where it must be kept by such additional doses as the exigencies of the case demand.

As an anodyne in these cases, we have found the valerianate of morphia the most satisfactory in its results. It is hardly necessary to say that the acetate of ammonia and stimulants are an indispensible part of our means of treatment in these cases, as well as those before mentioned.

But it is not only when the rash is imperfect that our patient may be found in an agony from nervous irritation. For a day or two after its full appearance the skin is in very much the same condition as if burned by the hot sun or with fire; and here also anodynes are often as indispensibly necessary. It is at this stage of the disease, and in the cases where the skin is dry and hot and no perspiration appears, that inunction is so very valuable, and it requires but little thought to perceive that exclusion of the air from the burned skin is the whole office of inunction. Such being the case the purest fresh lard is certainly the most proper article to use for this purpose. We consider bacon or any other sort of rancid or medicated grease as decidedly inferior.

The skin should be kept well protected by the lard till the rash begins to fade, or its necessity is superseded by a free perspiration; and we would here remark that we consider this or any other external application unnecessary and injurious in all cases where the skin is soft and the perspiration free. We see many cases in which, if properly treated, the rash goes through its course accompanied by a free perspiration and with little suffering from cutaneous irritation.

Especial care should be exercised to keep clear of anything which can interfere with the elimination of the materies morbi from the blood through the skin, and every action or neglect of action which can by any chance exert a derivative influence from the cutaneous surface should be carefully avoided.

To the use of emetics we have before alluded,—there are many cases where the operation of an emetic will rouse the system to throw out the rash, and have a decidedly beneficial influence; but we fear it is in the abuse of cathartic remedies that lies a greater mischief; the free operation of a cathartic at the forming stage of the disease can hardly fail, except in a decidedly phlogistic case, of cooling the surface and producing more or less retrocession of the rash. We would not be understood as sweepingly denouncing cathartics, but only their too frequent and indiscriminate use, for we have often seen the best effects from their proper administration at all stages, and are habitually using laxatives after the rash has faded and the danger of retrocession has passed away.

The external use of cold water has been recommended by good authority. We can readily understand that cases may occur in which the external application of water by sponging or the wet sheet of the hydropaths may prove beneficial, but with our views of the pathology of the disease we consider it a hazardous remedy. One of the worst cases we ever treated, was, we believe, rendered much worse than it would otherwise have been, by the free applibetion of cold water, previously to coming under our care.

Of the complications and sequelæ of this disease, our limits do not allow us to speak, nor is it fitting we should. Their treatment is in accordance with well known and established principles, with which all are or should be familiar.

The same application of general principles must guide us in the management of our patient during convalescence; for with the disappearance of the rash, after having run its perfect course, ends the specific nature of the disease, and any functional or organic lesion which then manifests itself, must be treated very much as if arising as an original disease. The deficiency and morbid state of the urinary secretion and the accompanying dropsy will usually yield to appropriate treatment; but it occasionally happens that a multiplicity of local mischiefs exhausts the already debilitated patient to so great an extent as to preclude recovery.

We take the liberty to present the following cases, which have but just passed from our hands, as examples of the way we treat the ordinary as well as the desperate cases of this disease.

M. D——, a lad 9 years, was attacked on Sunday, April 30th.—He vomited repeatedly during the day and night, the vomiting being accompanied by profuse diarrhœa. We first saw him on Monday, P. M. The evacuations had ceased and he was hot, dry, restless, with a slight appearance of rash about his neck and shoulders; pulse 140. He was ordered 3 grs. Dover powder every six hours, alternating with ℥ij. liquid acetate of ammonia, sinapisms to the feet, cold water to the head if agreeable, to be kept well covered in bed, and to drink freely of warm catnip tea.

Under this treatment the disease progressed favorably, and he was convalescent on Friday, May 5th. During the first three days of treatment the fever was intense, with a perfect eruption, and considerable delirium at night, yet he rested so well at intervals that it was not deemed advisable to make any application to the cutaneous surface, and on the third day (fourth of the disease) the skin became moist with an amelioration of all the symptoms. The bowels moved once a day during the sickness, and after he became convalescent without any aid from cathartic medicines. The throat was badly swollen, so much so as to render deglutation difficult; the ung. hyd. bin. iod. was applied externally, followed by a bread and milk poultice. Internally, solutions of nit. argent, tannin, and capsicum in vinegar, were applied at different times, and during convalescence it was gargled with strong alum water.

The family consisted of this lad and two sisters of the ages of five and twelve years, who were constantly about their brother from the first invasion till they in turn were attacked; the younger on Monday, May 8th, the other on Friday, the 12th.

The younger was attacked with vomiting, which continued with great severity some five or six hours, when profuse diarrhœa occurred, as in the first case. When first seen on Tuesday, P. M., she was stupid, with a very rapid feeble pulse, skin moderately dry and hot, but no appearance of the rash. The vomiting had ceased but the diarrhœa continued for some hours longer. A course of treatment similar to that pursued with the brother was followed for twenty-four hours, but with no good effect. At this time her pulse was 160, weak and fluttering, very slight appearance of rash, indifference to external impressions, was tossing about the bed to such an extent that several attendants were necessary to keep her covered, swallowed when anything was put in her mouth, but this could only be done by watching an opportunity to insert a few drops from a spoon between her lips, as she was rolling and tossing about.

Although the case appeared hopeless, we determined to persevere while there was life. She was put in a hot bath made by a large blanket dipped in hot water, in which she was kept enveloped for 20 minutes, (to do this required the united strength of two strong persons,) and 30 drops of tr. opii. were administered in divided doses within an hour, with but slight apparent effect. We then prescribed gr. 1-16 doses of valerianate of morphia, to be given every hour till she rested quietly, and then to be repeated as often as necessary, to keep her quiet, and to give a teaspoonful of liquid acetate of ammonia every hour, spirit to be added if she appeared cold or sinking.

The next morning, (May 10th, 8 A. M.,) the rash was fully developed , she had taken two doses of the morphia, and had rested conerably, but when not fully asleep was tossing about, and had no more general sensibility than the day before.

The medicines were continued, cold water applied to the head, and she was thoroughly greased with fresh lard every six hours.

8, P. M. Found her bed surrounded by mourning friends, waiting to see her expire, had taken no drink or medicine for six hours, as the parents thought she could not swallow. The incessant motion of her head, which could only be controlled by force, rendered it very difficult to get anything into her mouth. Taking her in our arms and holding her head still, we placed a cup of water to her lips ; she drank with avidity; after this drink and medicines were introduced in this way and without much difficulty. The symptoms from this time ameliorated, the anodyne was needed less frequently, but the acetate of ammonia was continued freely till the rash began to fade, on the 13th, at which time sensibility began slowly to return, and she to manifest her wants by words, and on the 15th she was decidedly convalescent. A diuretic, composed of tr. squills, tr. camphor opiata and spt. nitr. dulc., with occasional laxatives completed the cure.

The older girl was attacked as were the others by vomiting and purging, but less severely. These symptoms had ceased when we first saw her.

She was ordered 4 grs. dover powder every six hours, with ʒiij. doses of the acetate ammonia twice between, and to drink freely of catnip tea. Under this treatment, which did not require to be changed or modified for four days, the rash was perfectly developed, the skin never became dry, and she was convalescent on the 17th, having had a very mild run of the disease.

In closing we may be allowed to offer some suggestions which occur to us.

Some seem to regard the rash too much as an acute inflammation of the cutaneous tissue. From any such view we would dissent, for we can by no means acknowledge the eruption of this disease or that of Rubeola on Typhoid fever to be the result of inflammatory action. We consider it an effort of nature to throw off a materies morbi from the blood, and the subsequent desquamation of the cuticle as a result of morbid development of epithelial cells rather than a consequence of pure inflammation.

General blood-letting would be practised by no sane man except in such cases of plethora as are rarely found; we have never bled and never expect to.

The assertion that opium "tends to close up the outlets by which the morbid matter must be removed," we consider altogether too sweeping. That it diminishes the action of the bowels no one will deny, but if our fear of revulsion is well founded, this is a gain rather than a loss, and we have always been taught and yet believe that opium tends to promote increased cutaneous elimination, which is in our view the very object sought in this disease.

The local treatment of the throat should be governed by the application of those principles which are supposed to guide the practice of all educated physicians.

External stimulents and local internal applications are invaluiable when applied by a judicious hand. While one throat will be most benefitted by the "antiphlogistic contact" of nitrate of silver, another will need to be roused to healthy action by the tincture of capsicum, and both may need gargles of tannin or alum at a later stage, and he who cannot distinguish whether his patient needs either or neither of these, ought not to treat Scarlatina.

When an external counter irritant is needed, we prefer the ung. hyd. bin. iod., followed by a big bread and milk poultice. This will rarely if ever cause the unhealthy sores so liable to follow the use of cantharides in eruptive fevers.—*N. Hamp. Jour. Med.*

